# Supplementary material for: Are carbon emissions trading and green financial instruments synergistic? -Comprehensive quantitative research based on content analysis
Source: PLoS One. 2024 Mar 7;19(3):e0298601. doi: 10.1371/journal.pone.0298601 (PMC10919601; doi:10.1371/journal.pone.0298601)
Supplement: S1 Data — (ZIP) [file pone.0298601.s001.zip › Data/地级市Z_F工作报告.zip!/╝¬┴╓/╦─╞╜╩╨/└┤╫╘╣1⁄2╓┌║┼í╛┬φ┐╦╩2╛▌═°í┐_2007.docx]

各位代表：

　　现在，我代表四平市人民政府，向大会报告政府工作，请予审议。

　　一、“十一五”实现良好开局，经济和社会事业取得新成绩

　　2006年，全市生产总值完成380亿元，同比增长13.8%；一、二、三产业增加值分别完成138.8亿元、119.7亿元、121.5亿元，同比分别增长10%、18.8%、13.5%；全社会固定资产投资完成144.3亿元，同比增长65.1%；社会消费品零售总额实现128亿元，同比增长16.7%；一般预算全口径财政收入完成22.1亿元，同比增长15.4%，其中市区财政收入完成11.5亿元，同比增长17.9%。

　　——项目建设取得新成果。全市共开发建设项目612个，投资1000万元以上项目261个，其中亿元以上项目77个，完成投资126亿元，同比增长80%。昊华离子膜烧碱、天成玉米深加工、通钢冷轧板、双辽福耀玻璃、伊通金圆水泥等重点项目相继投产。振兴老工业基地第一、二批15个国债项目全部开工，完成投资15.1亿元。争取开行贷款到位资金6.86亿元。积极开展域外招商，参加第二届东北亚投资贸易博览会等大型招商活动,共引进50万元以上项目829个，到位资金100.3亿元。新加坡新达集团、中粮集团、香港立国集团等大企业先后到四平投资。全市外贸出口总额实现6847万美元，同比增长120.8%。开发区、工业集中区对外开放作用逐步增强。

　　——工业运行态势良好。全市规模以上工业企业完成增加值85亿元，同比增长20.9%；实现利润5.2亿元，同比增长52.2%，达历史最好水平。实施较大技改项目78个，完成投资35亿元，同比增长20%。加快中小企业服务体系建设，成立了中小企业服务中心、中小企业协会和行业协会，全市民营企业达到3073户，规模以上民营工业企业完成增加值51.5亿元，同比增长28.4%。

　　——农村经济全面发展。粮食产量达124.4亿斤，创历史新高。畜牧业发展势头强劲，猪、牛、羊、禽饲养量分别达到840.7万头、247.6万头、175.5万只、1.47亿只。农副食品加工业不断壮大，规模以上农副食品加工企业实现产值43亿元，同比增长10.9%。培训农民80万人，转移农村劳动力45万人次。农村合作经济组织发展到581个，带动农户7.6万户，提高了农民组织化程度和农产品竞争力。新农村建设试点工作稳步推进。

　　——城市建设水平不断提高。棚户区改造采取政府主导和市场化运作的办法，共拆迁旧房55.07万平方米，动迁居民9775户，建设楼房68.12万平方米。城市基础设施进一步完善，修建了六孔桥路、北邮电街、幸福路等9条主次干路，改造巷路23条。热网北干线延伸工程全面竣工，城市集中供热能力达到870万平方米。实施市区24小时供水改造工程，3万户居民受益。城市出口建设实现突破，四叶一级路、102线四平连接线竣工。环城公路基本完工，通车里程46.5公里。京哈铁路四梨路公铁立交桥实现简易通车。完成交通建设投资10亿元，竣工里程661.1公里。北河综合整治工程全面完成，修建滨河路10公里，滨河公园绿地28万平方米，园林广场8处。铁东公园、宏泰公园、热电广场及周边绿化工程全部竣工。创建国家园林城市开局顺利，新增绿地63.5万平方米。亮化工程取得阶段性成果，市区8个广场、23条街路、107个楼体实现了亮化。集中开展市区环境整治行动，国家卫生城市成果进一步巩固。

　　——群众生活继续改善。全年创造就业岗位5.77万个，新增就业4.9万人。全市共拨付养老金、失业救济金6.5亿元，确保了按时足额发放。发放小额担保贷款1646万元。城镇低保人数达到11.5万人，发放低保金9226万元。纳入农村低保8.8万人，发放低保金2113万元。建成62个农村福利中心，“五保户”供养条件有所改善。城镇居民人均可支配收入达到9900元，同比增长14.5%；农民人均纯收入达到3690元，同比增长13%。积极开展城市居民住院医疗保险，已参保2.9万人，其中低保对象0.4万人。加强食品、药品、农资等重点领域市场监管。全面规范企业用工行为，维护劳动者合法权益。积极开展扶贫帮困和灾民救济，困难群众得到及时救助。

　　——社会事业协调发展。走科技兴市之路，全市又有35个项目列入国家、省科技发展研发计划。基础教育不断加强，教育质量不断提高。事业单位人事制度改革稳步推进。卫生事业健康发展，新型农村合作医疗试点进展顺利，疾病预防控制体系和卫生监督体系进一步完善。竞技体育成绩显著，获得第十五届省运会夏季项目奖牌总数、金牌总数和团体总分第二名。广播电视基础设施不断完善，节目播出质量有所提高。文化艺术事业健康发展，网吧专项治理取得阶段性成果，四平战役纪念馆初步具备开馆条件。人口和计划生育工作取得新进展。1%人口抽样调查工作顺利完成。不断加大国有土地有偿使用力度，国土资源管理加强。环境污染得到有效控制，二龙山水库污染综合治理取得实质性突破。整顿规范房地产交易秩序初见成效，房地产交易中心投入使用。加大安全生产监管力度，全年未发生重特大安全事故。深入开展平安四平创建工作，社会治安形势稳定。双拥活动深入开展，军政军民关系进一步密切。精神文明和民主法制建设进一步加强。金融、知识产权、民族宗教、侨务、旅游、人防、地震、气象、老龄、档案、地方志和残疾人等各项工作取得新的成绩。

　　——政府自身建设得到加强。切实加强政府系统廉政建设，全面推进源头治腐工作。坚持依法行政，进一步清理行政许可项目、压缩各类项目审批时限。深入开展政务公开、效能监察和审计监督，纠正部门和行业不正之风，查办一批违法违纪案件。认真解决群众关心的热点、难点问题，妥善处理群众来信来访。自觉接受人大法律监督、工作监督和政协民主监督。全年办理人大代表议案和建议134件，政协委员提案182件，办复率达100%。

　　各位代表，这些成绩的取得，是市委正确领导的结果，是全市各族人民共同努力的结果，是社会各界大力支持的结果。在此，我代表市人民政府，向奋战在全市各条战线的广大劳动者，向给予政府工作热情支持的人大代表、政协委员、各民主党派、工商联、各人民团体和社会各界人士,向关心帮助四平建设的驻平部队、武警官兵、中省直单位及海内外朋友表示崇高的敬意和衷心的感谢！

　　总结工作，我们清醒地看到，我市经济和社会事业发展中还存在一些矛盾和问题，主要是经济总量不大、结构不优、财政困难，城镇居民收入较低；农业基础设施薄弱，农民增收渠道不宽，农业综合生产能力还需要进一步提高；就业再就业、社会保障压力较大，困难群众生产生活问题需要下大力气解决，维护社会稳定和化解矛盾的任务繁重；部分干部思想观念、工作作风与新形势、新任务的要求还不适应，这些问题需要在今后的工作中认真解决。

　　二、突出工作重点，全力推进经济和社会事业大发展快发展

　　2007年是“十一五”规划承前启后之年。今年工作的总体要求是：以邓小平理论和“三个代表”重要思想为指导，以科学发展观统领经济和社会发展全局，以市第五次党代会提出的早日实现四平振兴、人民富裕为目标，继续坚持工业立市、项目带动战略，把大上项目、全民招商作为突破口，坚持速度、质量、效益相协调，着力推动全市经济大发展、快发展，着力解决好民生问题，促进社会和谐稳定和全面进步。

　　主要预期目标是：全市完成生产总值460亿元，增长21%；万元生产总值能耗下降6%；一般预算全口径财政收入25.7亿元，增长16%；全社会固定资产投资220亿元，增长52.5%；城镇居民人均可支配收入实现11380元，农民人均纯收入4200元，分别增长15%和13.8%；新增城镇就业5万人，城镇登记失业率控制在4.5%以内；居民消费价格指数为103%；外贸进出口总额8000万美元以上；人口自然增长率控制在5‰以内。

　　上述指标是根据我市目前基础条件和发展状况确定的。由于我市经济总量小，人口多，即使完成上述指标，我们的人均主要经济指标在全省位次仍然靠后。最近几年，全省各地经济发展速度很快，不进则退，慢进也是退，我们必须进一步增强危机感、紧迫感和责任感，主动加压，奋力拼搏，采取超常规措施提速增效，确保完成或超额完成任务，推进四平大发展、快发展。为此，重点做好以下工作：

　　（一）狠抓工业，提高整体发展水平。全面提高企业自主创新能力，组织引导企业依托大专院校、科研单位，加强技术改造和新产品开发，提高装备技术和工艺水平。强化质量意识，大力培育和发展名牌。推进企业节能降耗，减排污染，切实转变增长方式。支持和帮助企业做大做强，引导生产要素向红嘴集团、四平烟厂、双辽发电厂、四平热电厂、黄龙、昊华、天成等大企业集聚，加快发展大型企业集团，打造产业集群，培育支柱产业。对规模以上企业，特别是50户重点企业，加强协调、调度，保证满负荷生产，实现增产扩销、提速增效。抓好亏损百万元以上企业的控亏、减亏和扭亏增盈。加强对中小企业的扶持，促进成长型中小企业上规模、上水平。关心、爱护、支持、保护企业家，建立健全培养、引进、使用和奖励机制，提高企业家素质，重点培养一批企业领军人物。

　　（二）强化农业，扎实推进新农村建设。以发展现代农业、增加农民收入为核心，加快新农村建设步伐。稳定粮食生产，加强农业基础设施建设，推广应用新品种、新技术，不断提高粮食综合生产能力。加快发展特色农业，一村一品，一乡一业，壮大产业规模。大力发展精品畜牧业，建设好专业化、规模化和标准化牧业小区，打造牧业大产业。加快发展农畜产品深加工业，培育龙头企业，延长产业链条，提高附加值。大力发展各类协会和专业合作经济组织，提高农业市场化水平。搞好农民职业技能培训，发展劳务经济，加快农村富余劳动力转移。搞好河流治理和防洪工程，强化水资源管理，启动二龙山水库水源地生态保持工程。抓好生态林业建设，保护林业资源。深入实施县域突破战略，全面提升县域经济实力。加快完善小城镇功能，不断提高辐射能力。深化农村各项改革，进一步做好农垦企业剥离办社会工作，清理农村“三角债”，化解债务链。抓好第二批新农村建设试点，积极发展农村公益事业，改善农村生产生活条件，争取新增100个行政村通油路（水泥路），竣工里程700公里。

　　（三）大上项目，拉动经济快速发展。把2007年作为“项目年”，牢固树立经济工作项目至上、项目压倒一切思想。重点上能够拉动地方经济发展的工业项目，上高科技含量、高附加值项目，上农副产品精深加工项目，上有利于扩大就业的劳动密集型项目，上资源循环利用生态环保型项目，上有利于增加财政收入的税源型项目。按照国家产业政策导向，组织专门力量，科学谋划，捕捉信息，筛选项目，搞好论证，提高项目成功率。紧紧抓住国家扶持东北老工业基地和粮食主产区产业化经营的机遇，积极向上争取项目。发挥我市能源、资源、土地、劳动力等优势，加强市场化运作，盘活闲置厂房、设备、场地等资产，吸引域外资本来平发展。以红嘴为样板，加快项目建设速度，加强对双辽发电厂、四平热电厂、昊华、天成、中粮、新达、新天龙等新建、改建、扩建大项目的协调、扶持和帮助，尽早开工建设，投产达效。进一步加大对开发区支持力度，加快开发区建设步伐，完善基础设施，打造最优环境，充分发挥开发区承载项目功能，用主要精力开发项目，真正把开发区和工业集中区建成项目园区。实行高位运作，加强调度、协调和服务，加大督查考核力度，强力推进项目建设实现新突破。

　　（四）全民招商，增强经济发展后劲。放手发动群众，形成全民招商、全社会招商的浓厚氛围。全年完成招商引资任务150亿元。将“长三角”、“珠三角”和环渤海地区，港澳台，韩国、日本、俄罗斯，东南亚作为招商重点区域，瞄准国内外战略投资者、大企业集团开展招商。积极参加第三届东北亚投资贸易博览会和“中俄国家年”等经贸交流活动。充分利用企业熟悉市场、掌握信息、联系广泛的优势，发挥企业招商主体作用，以商招商，把企业推向招商引资主战场。注重实际效果，创新招商方式，多开展小型化招商、专业化招商、网上招商、中介招商，依托驻外机构招商，降低成本，提高成功率。高度重视金融部门引进资金作用，及时掌握国家资金投向和扶持重点，改善金融环境，加强银企对接，通过地方金融部门牵线搭桥，积极争取上级和域外资金。进一步完善并严格兑现招商引资奖励政策，对招商引资有功人员，大张旗鼓进行表彰、奖励。围绕招商引资鼓实劲、想实招、求实效。落实招商引资责任制，着力解决各级干部动力不足、压力不够的问题。真正形成人人肩上有压力，齐心协力搞招商的局面。

　　（五）激发活力，加快发展民营经济。积极组织实施民营经济腾飞计划，破除不利于民营经济发展的各种障碍，实行“非禁即入”。激发全民创业活力，引导群众把手中资金变成创业资本。树立创业典型，发挥能人效应，继续鼓励机关、事业单位工作人员离岗领办、帮办、创办企业，鼓励城市有能力有实力的人员自主创业，鼓励农村各类能人创办实业。着力破解制约民营经济发展的突出问题，在资金融通、人才培养、信用担保、技术支持和管理咨询等方面提供有效服务。加快国有商业企业改制步伐，鼓励民营经济参与商贸、流通、服务等第三产业发展。利用区位优势和人文优势，大力发展物流业和旅游业。在加强对民营经济支持和保护的同时，引导民营企业和个体工商户提高自身素质，依法经营，履行社会责任。

　　（六）优化环境，打造发展竞争优势。增强发展意识、大局意识和服务意识，宁可牺牲眼前利益和局部利益，也要为投资者和纳税人创造更优环境。凡是国家、省、市已经出台的优惠政策，一定兑现。有些政策没有明确的事情，一事一议，特事特办。主动上门服务，为企业排忧解难，凡是能办的事情马上办。继续办好政务大厅，简化审批手续，提高办事效率，加快推进行政审批权相对集中改革，严格实行一站式办公，保证所有审批手续在政务大厅一次办结。公开行政事业收费项目和最低收费标准，实行一个窗口收费。定期或不定期征求投资者和纳税人的意见，让投资者和纳税人评判执法执罚和经济管理部门服务的好坏。严禁乱检查、乱收费、乱罚款、越权执法、吃拿卡要，对破坏经济发展软环境的行为，坚决从严查处，实行责任追究，公开进行曝光，不搞下不为例。

　　（七）建管并重，提升城市整体品位。高起点、高品位、高水平编制新的城市总体规划。在建设上本着量力而行、尽力而为的原则，重点抓好八件事：一是继续推进棚户区改造，完成省下达任务，妥善做好动迁居民回迁工作。全面启动县（市）棚户区改造工程。二是加强生态环境建设，创建国家级园林城市，力争年底实现创建目标。三是6月底前完成南北河蓄水工程，续建六孔桥上游南河改造工程。四是完成体育场征地、拆迁工作，着手建设新体育场。五是开工建设生活垃圾无害化处理场，实现污水处理厂达标运行。六是新建东山大街、河南路、南迎宾街，续建六孔桥路、北邮电街、北体育街、大顺街，同时，搞好主次街路维修。七是完成四梅铁路平东公铁立交桥和京哈铁路四梨路公铁立交桥工程。八是开工建设伊通至辽源高速公路四平段工程，完成国道102线四平至长春段一级路工程。同时，加快城市公用设施建设，改造供水管网，完善供热管网。加大城市管理力度，强化对交通秩序、主次街路和居民小区卫生、城市基础设施管护的治理整顿，进一步巩固国家卫生城市成果。加强养成教育，让每一个市民都能爱护城市，参与建设、管理城市。

　　（八）统筹兼顾，促进社会全面进步。继续实施科教兴市和人才强市战略，加大科技创新力度，构建产学研相结合的科技创新体系。加强人才队伍建设，制定奖励措施，加快培养和引进高层次人才，建立高层次人才库。注重对自主知识产权的开发、利用和保护，培育优势品牌。全面实施素质教育，切实加强基础教育，提升职业教育水平，积极发展民办教育和高中阶段教育，支持发展高等教育。大力开展全民健身活动，提高竞技体育综合实力。继续完善城乡公共卫生体系，加强城市社区卫生工作，加快建设农村三级卫生服务网络。抓好计划生育，稳定低生育水平，提高出生人口素质。积极发展文化事业，开发文化产业，加强文化市场管理。组织实施“广播电视村村通”工程，扩大广播电视覆盖面。严肃查处土地违法案件，保证重点项目建设用地。整顿房地产市场交易秩序，做好一次性处理无籍房工作。加强环境保护工作，打击环境违法行为。加强税收征管，堵塞漏洞，清缴欠税，做到应收尽收，增强财政支撑能力。树立“过紧日子”思想，厉行节约，把有限财力用在刀刃上。做好“双拥”工作，巩固双拥模范城成果。继续做好民族宗教、档案管理、供销、外事、侨务、气象、地震、人防、老龄、地方志等工作。

　　（九）关注民生，搞好和谐四平建设。进一步落实劳动者自主择业、市场调节就业和政府促进就业的方针，加强就业培训和职业介绍，办好就业孵化基地，落实税费减免、灵活就业人员社会保险补贴和小额担保贷款等优惠政策，扶持就业困难人员创业。搞好公益性岗位开发，千方百计增加就业岗位，着力解决好零就业家庭就业问题。继续扩大养老保险和失业保险覆盖面，确保养老金和失业救济金及时足额发放。进一步完善城乡居民医疗保险制度，加快推进城镇居民基本医疗保险，全面开展新型农村合作医疗试点。加快建立城乡社会综合救助体系，实现城乡最低生活保障全覆盖，保障灾民和困难群众生产生活。大力发展社会福利事业，完成农村社会福利中心建设任务。按照“属地管理，分级负责，谁主管谁负责”原则，落实信访责任制，变上访为下访，做好矛盾纠纷排查调处，把问题解决在基层和萌芽之中。妥善处理工业企业改制遗留问题，积极推进厂办大集体企业改革试点。切实抓好安全生产，有效防范和化解各种突发事件。整顿和规范市场经济秩序，强化食品、药品安全监管，坚决惩处制假售假和商业欺诈等行为。深入开展平安四平创建工作，加强治安防控体系建设，依法严厉打击各种刑事犯罪活动，增强人民群众安全感，维护社会稳定。团结一切可以团结的力量，调动一切积极因素，营造良好风气，建设和谐四平。

　　三、加强政府自身建设，为大发展快发展提供保证

　　推动四平大发展、快发展，必须转变政府职能，建设亲民、高效、诚信、廉洁、法治政府。

　　建设亲民政府。坚持以民为本，把群众满意不满意作为一切工作的根本出发点和落脚点，时刻想着人民，经常深入群众，了解群众疾苦，设身处地为群众着想。更加关注弱势群体，多做雪中送炭的事情，多办顺应民意、化解民忧、为民谋利的实事，切实解决好群众最关心、最直接、最现实的利益问题，让群众年年都能真正感受到发展成果。

　　建设高效政府。向红嘴学习，增强事业心、责任感，以对党和人民高度负责的精神，解放思想，敢为人先，恪尽职守，真抓实干，勇于负责，敢于碰硬，集中攻坚克难。加快工作节奏，提高行政效能，确保政令畅通，做到令行禁止。切实加强学习，全面提高公务员队伍素质，提高工作能力和服务水平。大力精简会议和文件，多深入基层，多解决实际问题，把主要精力放在抓落实上。对重点工作，要持之以恒，一抓到底，不达目的不罢休。

　　建设诚信政府。坚持言必信，行必果。不图虚名，不做表面文章，说实话，干实事，求实效。办不到的事情不说，说了就一定努力办到。以诚待人，以信取胜，提高政府公信力。凡涉及经济社会发展、群众利益的重大决策，都要经过充分调查、论证，反复征求各方面意见，科学决策、民主决策，经得起历史检验。

　　建设廉洁政府。全面落实廉政建设责任制，严格执行廉洁从政的各项规定，建立健全教育、制度、监督并重的惩治和预防腐败体系，做到警钟长鸣。牢记“两个务必”，努力为人民掌好权、用好权，决不以权谋私。领导干部带头管好自己、家属子女和身边工作人员，清正廉洁，干净干事。深入推行政务公开，增强政府工作透明度，铲除滋生腐败的土壤。严肃查处各种违法违纪案件，深入开展治理商业贿赂工作。加强专项治理，坚决纠正部门和行业不正之风。

　　建设法治政府。坚持依法行政，依法管理经济和社会事务，严格按照法定权限行使行政权力，履行职责。完善行政复议制度，规范行政执法行为。自觉接受人大的法律监督、工作监督和政协的民主监督，接受社会公众和新闻舆论监督。认真办理人大议案、建议和政协提案，加强同各民主党派、群众团体、无党派人士的联系，充分听取社会各界的意见和建议，不断改进政府工作。

　　各位代表！四平是一座英雄的城市，现在正处在加快发展的关键时期。让我们在市委领导下，开拓进取，奋力拼搏，一心一意谋发展，集中精力抓落实，全面完成今年经济和社会发展的各项任务，着力推进大发展、快发展，着力解决好民生问题，为早日实现四平振兴，人民富裕而努力奋斗！
